# The role of *IL17* and *IL17RA* polymorphisms in lethal pandemic acute viral pneumonia (Influenza A virus H1N1 subtype)

**DOI:** 10.1186/s42047-023-00126-0

**Published:** 2023-02-08

**Authors:** Vanessa Yumie Salomão Watanabe Liberalesso, Marina Luise Viola Azevedo, Mineia Alessandra Scaranello Malaquias, Caroline Busatta Vaz de Paula, Seigo Nagashima, Daiane Gavlik de Souza, Plínio Cézar Neto, Kauana Oliveira Gouveia, Larissa Cristina Biscaro, Ana Luisa Garcia Giamberardino, Gabrielle Tasso Gonçalves, Thais Teles Soares Kondo, Sonia Maria Raboni, Isabelle Weiss, Cleber Machado-Souza, Lucia de Noronha

**Affiliations:** 1 Postgraduation Program in Health Sciences of School of Medicine, Pontifícia Universidade Católica Do Paraná, Curitiba, Brazil; 2 Laboratory of Virology, Hospital de Clínicas, Universidade Federal Do Paraná, Curitiba, Brazil; 3 Postgraduation Program in Biotechnology Applied in Health of Children and Adolescent, Faculdades Pequeno Príncipe, Curitiba, Brazil

**Keywords:** Influenza A virus H1N1 subtype, Influenza, Immunohistochemistry, Polymorphism, *IL17A*, *IL17RA*

## Abstract

**Background:**

The cytokines play an essential role in acute inflammatory processes, and the IL-17 may be responsible for ambiguous aspects, and the correlation with genetic polymorphisms could improve the search for this critical biomarker. Thus, this study aimed to evaluate the IL-17A and IL-17RA tissue expression and the polymorphisms that codified these proteins in a population that died of pandemic Influenza A virus H1N1 subtype compared to a non-pandemic Influenza virus population.

**Methods:**

Necropsy lung samples immunohistochemistry was performed to assess the presence of IL-17A and IL-17RA in the pulmonary tissue. Eight single nucleotide polymorphisms were genotyped using TaqMan® technology.

**Results:**

The Influenza A H1N1 pandemic group had higher tissue expression of IL-17A, higher neutrophil recruitment and shorter survival time between admission and death. Three single nucleotide polymorphisms conferred risk for pandemic influenza A H1N1, the AA genotype of rs3819025 G/A, the CC genotype of rs2241044 A/C, and the TT genotype of rs 2,241,043 C/T.

**Conclusions:**

One IL17A polymorphism (rs381905) and two IL17RA polymorphisms (rs2241044 and rs2241043) represented biomarkers of worse prognosis in the population infected with pandemic influenza A H1N1. The greater tissue expression of IL-17A shows a Th17 polarization and highlights the aggressiveness of the pandemic influenza virus with its duality in the protection and pathogenesis of the pulmonary infectious process.

## Background

Lower respiratory infections reach the extremes of age and account for many deaths in children under five years of age (Jain et al*.,* 2015a; Jain et al., 2015b; Unicef, 2021). Viruses are responsible for many respiratory infections and morbidities, supported by the lack of specific therapeutic measures (Pavia, 2011; Jain et al., 2015a). Among the viruses associated with pneumonia, respiratory syncytial virus, human rhinovirus, human metapneumovirus, adenovirus, parainfluenza, influenza A (FLUA) and B, coronavirus stand out (Jain et al*.,* 2015a). Analyzing cases of community-acquired pneumonia that required hospitalization, 45% occurred in children under two years of age and 21% of children up to 18 years of age required intensive care. The most frequent etiological agent was viruses (Jain et al., 2015a).

Influenza viruses, members of the Orthomyxoviridae family, are important respiratory pathogens that can cause sporadic cases, seasonal epidemics, and periodic pandemics that impact human health and the economy (Krammer et al. 2018). The disease involves symptoms such as fever, coryza, cough, odynophagia, myalgia, headache, and self-limiting fatigue. It can evolve, compromising the lower airways, causing bronchiolitis, pneumonia, acute respiratory distress syndrome and predisposing to secondary bacterial infection (Krammer et al. 2018; Clementi et al. 2021). A pandemic is possible when a new strain of Influenza of zoonotic origin antigenically different from the circulating strains appears (Krammer et al. 2018).

The most recent FLUA virus pandemic occurred in 2009 with serotype H1N1 (H1N1pdm09), a triple recombinant in human, swine and avian Influenza (Kasowski et al., 2011). This virus was detected in Mexico between February and March, and in California, it was confirmed in April. On April 25, 2009, the World Health Organization declared an international public health emergency (CDC, 2009). In epidemiological terms, this pandemic was highlighted by the mortality among children and young adults (Kasowski et al., 2011). H1N1pdm09 differs from the seasonal FLUA in inducing pro-inflammatory responses in human and animal models (Itoh et al. 2009). This virus has distinct clinical-pathological characteristics and is associated with the involvement of other organs, such as the central nervous system and myocardium (Glaser et al*.,* 2012). Lung injury leads to diffuse alveolar damage, causing more complications such as hypoxemia and admissions to intensive care units than the seasonal influenza virus (Lee et al*.,* 2011). There is an excessive host immune response with high production of the most diverse inflammatory mediators (Lee et al*.,* 2011; Bal et al. 2012; Clementi et al. 2021).

The severity of H1N1pdm09 infection associated with hospitalization shows an increase in inflammatory mediators associated with Th17 and IL-17A production compared to mild clinical manifestations (Bermejo-Martin et al*.,* 2009). When analyzing inflammatory mediators and outcomes, higher serum levels of IL-17A collected 24 h after hospital admission was associated with a higher 28-day survival rate (Almansa et al. 2011). In addition, FLUA infection is related to increased expression of IL17-A/IL-17RA in lung tissue (Azevedo et al*.,* 2020). There is a duality between the protective and pathological function of IL-17A and single nucleotide polymorphisms (SNPs), which can modify the evolution and outcome of FLUA virus infection (Bermejo-Martin et al., 2009; To et al. 2010; Sahu et al. 2021).

IL-17A was described in 1993 and named CTLA-8 (cytotoxic T lymphocyte-associated protein 8) (Rouvier et al. 1993). The IL-17A family is represented by six interleukins that signal as homodimers (IL-17A, IL17B, IL-17C, IL17-D, IL17-E), in addition to the IL-17A/IL17-F heterodimer (Goepfert et al. 2017; Monin & Gaffen 2018). They are produced by Th17 cells (Rouvier et al. 1993).

These interleukins activate their signaling through five receptors (IL17-RA, IL17-RB, IL17-RC, IL17-RD, IL17-RE) (Monin & Gaffen 2018). Immunity measured by IL17-A/IL-17RA can interfere with host defense in the acute and chronic phases of lung inflammation through mechanisms related to pathogen load limitation and tolerance, demonstrated in bacterial infection models (Monin & Gaffen 2018). It is a pro-inflammatory cytokine that stimulates the expression of IL-6 and participates in the pathophysiology of autoimmune, infectious, inflammatory and cancer diseases (NCBI, 2020a). The *IL17A* gene encoding this protein is located at 6p12.2. The *IL17RA* gene is located at 22q11.1 and encodes a membrane glycoprotein that binds to IL-17A (NCBI, 2020b).

Genetic biomarkers may indicate specific alleles associated with the pathogenesis of infectious diseases. Thus, variations in the nucleotide sequence, that is, genetic polymorphisms, can modify the amino acid composition of a protein, causing changes in its functions and consequent changes in the pathophysiology and functioning of a target organ (Chanock et al*.,* 2001). Genetic variants can influence the resistance to infection, relating to the mechanisms of virus entry into the cell, the over or underproduction of cytokines and the alteration of the innate and adaptive immune response (Kenney et al. 2017; Pérez-Rubio et al. 2021).

The association between a disease and its complications with tissue and genetic biomarkers, in addition to allowing the identification of susceptible individuals, contributes to elucidating factors related to the evolution and outcome of the disease (Pérez-Rubio et al. 2021). In this context, tissue levels of IL-17A seem to actively participate in the pathogenesis of lung injury induced by viruses, especially H1N1pdm09, due to its great capacity for leukocyte recruitment and maintenance of the inflammatory response (Fajgenbaum & June 2020).

This study investigates whether polymorphisms in the *IL17* cluster gene (*IL17A* and *IL17RA*) are associated with increased tissue expression of IL-17A and IL-17RA proteins in patients who died of H1N1pdm09.

## Materials and methods

### Ethical approvals

The ethics review board of the HC-UFPR reviewed and approved the study (register numbers: 1099.138/2005; 76,401,717.0.0000.0096/2017). The *families authorized the post-mortem* pulmonary biopsy, and the consent forms were informed and signed. The individuals who participated as a negative control had their consent forms informed, signed, and approved by the Human Research Ethics Committee of PUCPR (No. 25141813.4.0000.0020).

### Databases

Three groups were composed for this study (pandemic, non-pandemic and control).

Pandemic group: consisted of ten (10) patients who died of severe pulmonary disease due to pandemic Influenza A virus H1N1 subtype in 2009 (H1N1pdm09), autopsied at the Hospital de Clínicas—Universidade Federal do Paraná. The lung samples were separated, and formalin-fixed paraffin-embedded (FFPE) blocks containing lung tissue in good condition were used in the multi-sample blocks. The genotyping of H1N1pdm09 was performed by RT-qPCR (reverse transcriptase reaction followed by a polymerase chain reaction in Time Real). For amplification of the viral genome, the Kit Invitrogen SuperScriptTMIII Platinum® One-Step Quantitative RT-PCR.

Non-pandemic group: consisted of 30 FFPE samples. These samples were obtained from 193 pediatric cases involving necropsy (occurring between 1960 and 2004) and whose cause of death was lethal non-pandemic acute pneumonia (Baurakiades et al. 2014; Chong et al. 2009; de Souza Costa et al. 2014; do Carmo Debur et al., 2010). All 193 cases had already been tested for the presence of respiratory syncytial virus (RSV), adenovirus (AdV), parainfluenza type 1, 2 or 3 (PIV1, PIV2, and PIV3) and Influenza type A and type B (FLUA and FLUB) throughout immunohistochemistry (Azevedo et al., 2020; Baurakiades et al. 2014; Chong et al. 2009; de Souza Costa et al. 2014; do Carmo Debur et al., 2010). Sixty-eight cases tested positive for the virus. From these positive samples, cases of influenza A, B or both were selected.

Control group: was composed of 30 healthy individuals (only for the genotyping analysis) who sought the Dental Clinic of the Pontifícia Universidade do Paraná (PUCPR).

### Tissue Microarray (TMA) and Immunohistochemistry (IHC)

For the construction of tissue microarray blocks (TMA), two peri bronchial areas and two peripheral areas were punctured (3 mm samples from each case) and removed from the original blocks (donor blocks) organized in multi-sample paraffin blocks (recipient blocks).

One conventional slide from each TMA was stained using hematoxylin and eosin—H&E (Harris Hematoxylin: NewProv, Cod. PA203, Pinhais, BR; Eosin: BIOTEC Reagentes Analíticos, Cod. 4371, Pinhais, BR) and the other slides were separated for immunohistochemical study.

To observe the expression of de IL-17A and IL-17RA in the alveolar septa throughout immunohistochemistry, the primary antibodies for anti-IL-17 (rabbit polyclonal; 1:600 dilution from Bioss™) and IL-17RA (rabbit polyclonal; 1:400 dilution from Cloud-Clone Corp.) were used. All immunohistochemistry assays included a negative (omitting primary antibody) and positive control. An immunoperoxidase assay was performed in lung samples (immunohistochemistry). Antigen retrieval was performed using a BioSB™ ImmunoRetriever. Tissue samples were incubated with the primary antibodies (anti-IL-17A/IL-17RA) in a moist chamber at room temperature for one hour. Incubations with the secondary antibody (Dako Advance™ HRP System, DakoCytomation, Inc., CA, USA) were carried out for 30 min. Incubations with 3,3'-diaminobenzidine and hydrogen peroxide substrate (DakoCytomation, Inc., CA, USA) were performed for 3 min to visualize positive staining (Azevedo et al., 2020).

### Morphological analysis of protein expression

IL-17A and IL-17RA immunoexpression was assessed by quantitative analysis. Quantitative analysis (morphometry) was based on images obtained by the Axion Scan.Z1 Scanner slide scanner model (Carl Zeiss AG, Oberkochen, Germany).

The immunostained slides were digitized using the Axio Scan.Z1 Slide Scanner (Zeiss, Jena, Germany). Each digitized slide generates many images by the ZEN Blue Edition software (Zeiss, Jena, Germany), covering all samples. Images with no sample areas (blank images) or containing histological/immunohistochemical artifacts were discarded. All valid images after this first selection were submitted blindly and randomly discarded until only the desired number remained. This second selection is performed blindly, with random sampling regions, without the interference of the observer.

For each sample, approximately 600 images were obtained in a 20 × magnification objective, and of these, approximately 500 were excluded, generating 100 satisfactory images for analysis. For the positive control, the image containing adequate levels of tissue immunoexpression was chosen as the "mask." The mask was then superimposed on all sample images. The morphometric analysis was performed using color morphometry through the Image-Pro Plus 4.5 software (Rockville, MD, USA) (Malaquias et al. 2018). Immunopositivity areas in square micrometers of each photomicrograph were compiled and transformed into percentages (Azevedo et al., 2020).

Slides with hematoxylin–eosin were submitted to the Axion Scan.Z1 scanner (Carl Zeiss AG, Oberkochen, Germany) in a high-definition field to generate high-resolution images for neutrophil counts. The images were selected in the areas with the highest concentration of neutrophils (hot spots), with 10 images for each case. Counting was performed using the ZEN blue edition software (Carl Zeiss AG—Oberkochen, Germany), where each cell account was marked for further verification. Neutrophils found only in the septum, or alveolar lumen were marked. The average number of cells per sample was obtained to perform a comparative statistical analysis between the studied groups (Azevedo et al. 2021).

### Genetic extraction

Pandemic and non-pandemic groups were obtained from paraffinized samples' cuts using a commercially available paraffin DNA extraction kit (Qiagen®). After determining the concentration, the samples were diluted into a concentration of 20 ng/µl for working solution and stored in a freezer at –20ºC with restricted access and only allowed to researchers involved in the project or to the technical personnel for them authorized (Azevedo et al. 2021).

The Control group was obtained from saliva collected from patients to acquire buccal epithelial cells. The cells were submitted to centrifugation at 2000 rpm for 10 min for sedimentation. This process resulted in a supernatant, which was discarded, and the cell pellet resuspended in 1300 μL extraction buffer [10 mM Tris–HCl (pH 7.8), 5 mM EDTA, 0.5% SDS]. Ten microliters of proteinase K (20 mg/mL) were added to the solution, remaining overnight at 65ºC. DNA purification was performed by adding 10 M ammonium acetate, precipitated with isopropanol and resuspended with 50 μL of 10 mM Tris (pH 7.6) and 1 mM EDTA (Aidar & Line 2007). The obtained DNA was stored at -20 °C.

### Genetic amplification

The polymorphisms in the proposed genes were chosen by authors' criteria using functional relevance analysis obtained by significant results in papers published in qualified journals. In addition, it was verified on the SNPinfo site if the previously chosen SNPs were present. This website of the National Institutes of Health has a tagger SNP selection tool that occurs after the insertion of specific data, such as the name of the proposed gene and study population (SNPinfo, 2020). The patients' purified DNA was amplified by real-time PCR (Applied Biosystems 7500 Real-Time PCR System). The TaqMan system of allelic discrimination is an assay in which genomic variants are detected through a multiplex polymerase chain reaction, which combines the amplification and detection of the polymorphic segment in a single step. Genotype determination was performed for the following *IL17A* polymorphisms: rs227513, rs3819025 and *IL17RA* polymorphisms: rs2241044, rs9606615, rs2241049, rs917864, rs879577, rs2241043.

### Statistical analysis

Immunohistochemical results were expressed as means, medians, minimum values, maximum values, standard deviations (quantitative variables), or absolute numbers and percentages (categorical variables). Spearman's correlation coefficient was estimated to assess the correlation between two quantitative variables. The comparison of groups concerning continuous quantitative variables that met the condition of normality was performed in the Student's t-test for independent samples (two groups) or the one-way analysis of variance (ANOVA) model (three groups). Variables that did not meet the normality condition were analyzed considering the non-parametric Mann–Whitney test. The Log-rank test was performed for survival time analysis, and Kaplan–Meier curves were presented. The normality condition of continuous quantitative variables was assessed using the Kolmogorov–Smirnov test. Values of *p* < 0.05 indicated statistical significance. For multiple groups, comparisons *of p* values were corrected by Bonferroni. Data were analyzed using the computer program Stata/SE v.14.1. StataCorpLP, USA.

## Results

### Patient's baseline

The sociodemographic and clinicopathologic characteristics of the study population are found in Table 1. The pandemic group showed higher tissue expression of IL-17A (*p* < 0.001) and neutrophil count (*p* = 0.001) when compared to the non-pandemic group.

**Table 1:** Baseline and Immunohistochemistry characteristics in the study population

Variables	Pandemic H1N1 (*n* = 10)	Non-Pandemic (*n* = 30)	*p*-value
**Age**^a^	43.5 ± 14.0	2.7 ± 3.3	< 0.001^c^
**Gender**^b^			
Male	8 (38.1)	13 (61.9)	0.069^d^
Female	2 (10.5)	17 (89.5)	
**Expression of IL-17A**^a^	9.9 ± 3.6	3.9 ± 3.2	< 0.001^c^
**Expression of IL-17RA**^a^	1.2 ± 0.9	3.3 ± 3.8	0.141^c^
**Neutrophil**^a^	17.6 ± 4.8	8.7 ± 8.5	0.001^c^
**Survival time (days)**^a^	4.7 ± 6.1	12.3 ± 6.3	0.019^c^

^a^Mean ± Standard Deviation

^b^Absolute number (percentage)

^c^Mann-Whitney U

^d^Pearson's chi-square test

### Genotyping results

To analyze the SNPs, three concepts were used (additive, dominant and recessive) concerning the transmission models. The additive model shows the distribution of genotypes. The dominant model groups the homozygous and heterozygous genotypes of the wild-type allele and compares them with the homozygous genotype of the polymorphic allele. The recessive model groups the homozygous and heterozygous genotypes of the polymorphic allele and compares it with the homozygous genotype of the wild-type allele. Table 2 shows the genotype results.

**Table 2:** Genotypic analysis of the tag SNPs in the *IL17A* and *IL17RA* gene in the addictive model

**Gene and reference SNP**^**a**^** Variation** **[1/2]**	**Groups**	**Homozygous** 1/1	**Heterozygous** **1/2**	**Homozygous 2/2**	*p*-value*
*IL17A*		**GG**	**GA**	**AA**	
rs3819025 [G/A]	Pandemic H1N1	N/A	2 (20.0)	8 (80.0)	
	Non-pandemic	1 (3.3)	7 (23.3)	22 (73.3)	0.587
	Control	1 (3.3)	28 (93.3)	1 (3.3)	**0.001**
*IL17A*		**GG**	**GA**	**AA**	
rs2275913 [G/A]	Pandemic H1N1	5 (50.0)	4 (40.0)	1 (10.0)	
	Non-pandemic	7 (25.9)	7 (25.9)	13 (48.2)	0.055
	Control	9 (31.0)	19 (65.5)	1 (3.5)	0.548
*IL17RA*		**AA**	**AC**	**CC**	
rs2241044 [A/C]	Pandemic H1N1	N/A	1 (11.1)	8 (88.9)	
	Non-pandemic	1 (3.5)	19 (65.5)	9 (31.0)	**0.012**
	Control	3 (10.0)	26 (86.7)	1 (3.3)	**0.001**
*IL17RA*		**TT**	**TC**	**CC**	
rs9606615 [T/C]	Pandemic H1N1	3 (30.0)	7 (70.0)	N/A	
	Non-pandemic	4 (13.8)	12 (41.4)	13 (44.8)	**0.027**
	Control	4 (13.3)	8 (26.7)	18 (60.0)	**0.01**
*IL17RA*		**AA**	**AG**	**GG**	
rs2241049 [A/G]	Pandemic H1N1	4 (57.1)	3 (42.9)	N/A	
	Non-pandemic	17 (63.0)	10 (37.0)	N/A	0.778
	Control	6 (20.0)	21 (70.0)	3 (10.0)	0.054
*IL17RA*		**TT**	**TC**	**CC**	
rs917864 [T/C]	Pandemic H1N1	3 (30.0)	5 (50.0)	2 (20.0)	
	Non-pandemic	2 (8.3)	12 (50.0)	10 (41.7)	0.102
	Control	N/A	9 (30.0)	21 (70.0)	**0.005**
*IL17RA*		**CC**	**CT**	**TT**	
rs879577 [C/T]	Pandemic H1N1	3 (30.0)	7 (70.0)	N/A	
	Non-pandemic	3 (16.7)	15 (83.3)	N/A	0.416
	Control	21 (75.0)	4 (14.3)	3 (10.7)	0.157
*IL17RA*		**CC**	**CT**	**TT**	
rs2241043 [C/T]	Pandemic H1N1	1 (10.0)	1 (10.0)	8 (80.0)	
	Non-pandemic	15 (71.4)	1 (4.8)	5 (23.8)	**0.005**
	Control	6 (20.7)	22 (75.9)	1 (3.5)	**0.001**

^a^SNP identifier based on NCBI dbSNP; Genotype was expressed by number and percentage, and a total percentage was shown in line

*N/A* Not applicable

^*^Logistic regression

### *IL17A* Polymorphisms

Rs3819025 G/A: 80% of the pandemic group individuals are homozygous for the polymorphic allele (AA) and presented statistical significance compared to the control group (*p* = 0.001).

### *IL17RA* Polymorphisms

Rs2241044 A/C: 88.9% of the pandemic group individuals are homozygous for the polymorphic allele (CC) and presented statistical significance when compared to the non-pandemic group (*p* = 0.012) and control group (*p* = 0.001).

rs2241043 C/T: 80% of the pandemic group individuals are homozygous for the polymorphic allele (TT) and presented statistical significance when compared to the non-pandemic group (*p* = 0.005) and control group (*p* = 0.001).

### Immunohistochemistry results

There was higher tissue expression of IL17A in the pandemic H1N1 group for all genotypes analyzed (Table 3).

**Table 3:** Correlation between tissue expression and genotyping distribution in *IL17A and IL17RA* for three models

Reference SNP^a^	Addictive model			Dominant model		Recessive model	
**rs3819025 G/A**	**GG**	**AG**	**AA**	**GG + GA**	**AA**	**AA + GA**	**GG**
Pandemic H1N1	N/A	10.3 ± 7.1	9.7 ± 3.0	10.3 ± 7.1	9.8 ± 3.0	9.9 ± 3.6	N/A
Non-pandemic	9.9 ± 0.0	3.4 ± 1.9	3.8 ± 3.4	4.3 ± 3.0	3.8 ± 3.4	3.7 ± 3.1	9.9 ± 0.0
**rs2275913 G/A**	**GG**	**AG**	**AA**	**GG + GA**	**AA**	**AA + GA**	**GG**
Pandemic H1N1	9.4 ± 3.8	10.3 ± 4.3	10.5 ± 0.0	9.8 ± 3.8	10.5 ± 0.0	10.3 ± 3.7	9.4 ± 3.8
Non-pandemic	4.8 ± 3.6	4.0 ± 3.8	4.2 ± 3.1	4.3 ± 3.6	4.2 ± 3.1	4.1 ± 3.3	4.8 ± 3.6
**rs2241044 A/C**	**AA**	**AC**	**CC**	**AA + AC**	**CC**	**CC + AC**	**AA**
Pandemic H1N1	N/A	2.9 ± 0.0	1.0 ± 0.8	2.9 ± 0.0	1.0 ± 0.8	1.2 ± 1.0	N/A
Non-pandemic	1.6 ± 0.0	2.6 ± 2.1	5.7 ± 6.0	2.6 ± 2.0	5.7 ± 6.0	3.6 ± 3.9	1.6 ± 0.0
**rs9606615 T/C**	**CC**	**CT**	**TT**	**TT + CT**	**CC**	**CC + CT**	**TT**
Pandemic H1N1	N/A	1.0 ± 1.0	1.6 ± 0.8	1.2 ± 1.0	N/A	1.0 ± 1.0	1.6 ± 0.8
Non-pandemic	4.6 ± 4.8	2.7 ± 2.9	1.9 ± 3.1	2.5 ± 2.9	4.6 ± 4.8	3.6 ± 4.0	1.9 ± 3.1
**rs2241049 A/G**	**AA**	**AG**	**GG**	**GG + AG**	**AA**	**AA + AG**	**GG**
Pandemic H1N1	1.0 ± 1.1	1.7 ± 1.4	N/A	1.7 ± 1.4	1.0 ± 1.1	1.3 ± 1.1	N/A
Non-pandemic	4.1 ± 4.5	1.5 ± 1.0	N/A	1.5 ± 1.0	4.1 ± 4.5	3.1 ± 3.7	N/A
**rs917864 T/C**	**CC**	**CT**	**TT**	**TT + CT**	**CC**	**CC + CT**	**TT**
Pandemic H1N1	1.7 ± 1.1	1.1 ± 1.1	0.9 ± 0.9	1.0 ± 0.9	1.7 ± 1.1	1.3 ± 1.0	0.9 ±0.9
Non-pandemic	1.6 ± 1.0	4.4 ± 4.9	1.1 ± 0.7	3.9 ± 4.6	1.6 ± 1.0	3.3 ± 4.0	1.1 ± 0.7
**rs879577 C/T**	**CC**	**CT**	**TT**	**TT + CT**	**CC**	**CC + CT**	**TT**
Pandemic H1N1	0.9 ± 1.4	1.3 ± 0.8	N/A	1.3 ± 0.8	0.9 ± 1.4	1.2 ± 1.0	N/A
Non-pandemic	3.6 ± 3.0	2.6 ± 2.8	N/A	2.6 ± 2.8	3.6 ± 3.0	2.8 ± 2.8	N/A
**rs2241043 C/T**	**CC**	**CT**	**TT**	**TT + CT**	**CC**	**CC + CT**	**TT**
Pandemic H1N1	2.9 ± 0.0	0.9 ± 0.0	1.0 ± 0.8	1.0 ± 0.8	2.9 ± 0.0	1.9 ± 1.4	1.0 ± 0.8
Non-pandemic	3.6 ± 4.4	N/A	1.7 ± 0.3	1.3 ± 0.9	3.6 ± 4.4	3.4 ± 4.3	1.7 ± 0.3

^a^SNP identifier based on NCBI dbSNP

^*^Mean ± Standard Deviation

*N/A* Not applicable

### Survival analysis

**Fig. 1:**
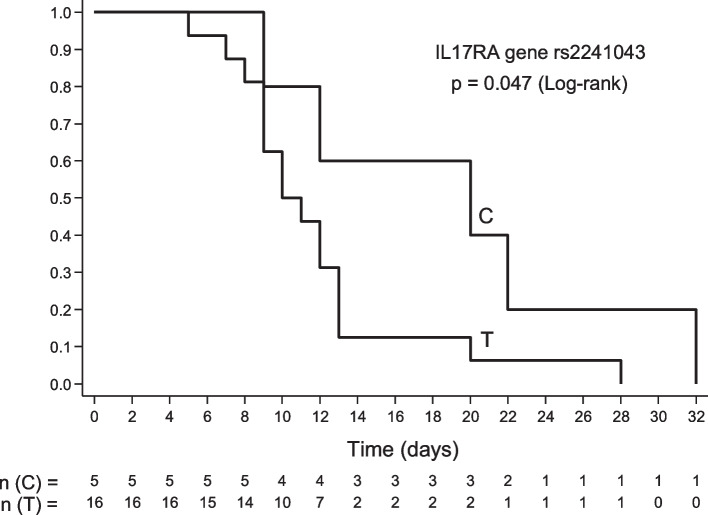
*IL17RA* gene rs2241043 (C/T) and association with longest survival time in the non-pandemic group concerning the C allele

The null hypothesis tested was that the survival time, which corresponds to the interval between hospital admission and death, is the same for both groups. However, there was evidence of the alternative hypothesis (*p* = 0.019), indicating the pandemic group had a shorter life span between hospitalization and death (4.7 ± 6.1) when compared to the non-pandemic group (12.3 ± 6.3). In addition, survival analysis was performed for each gene and their respective markers concerning the survival time. The *IL17RA* gene rs2241043 (C/T) indicated an association (*p* = 0.047) with the longest survival time (19.0 ± 9.1) in the non-pandemic group concerning the C allele (Fig. 1).

## Discussion

Individuals' susceptibility to disease has been attributed to specific alleles in risk-related genes. Genetic variations have also been suggested as possible susceptibility components to diseases caused by intracellular viral or bacterial pathogens. Studies of the human genome report direct associations between genetic polymorphisms and their relationship with susceptibility or resistance in many primary infectious diseases (Chapman & Hill 2012). The study investigated whether polymorphisms in the *IL17* cluster gene (*IL17A* and *IL17RA*) are associated with increased tissue expression of IL-17A and IL-17RA proteins in patients who died from H1N1pdm09.

Respiratory viruses compromise age extremes (Walter et al*.*, 2017; Schuster &Williams, 2018). H1N1pdm09 has its highest occurrence in young adults, with a lower incidence among older people over 65 years old (Kumar et al., 2010). The ancestor of hemagglutinin is the 1918 virus, which explains the lower impact on the population born before 1947 (Kasowski et al., 2011).

Tissue damage in FLUA infection results from the direct and indirect action of the virus (Clementi et al. 2021). Neutrophil chemotaxis and increased pro-inflammatory cytokines are among the leading causes of acute lung injury (Everitt et al*.,* 2012). In the pulmonary alveolus, FLUA directly infects pneumocytes II. Cells are recruited to the site of infection, such as macrophages, neutrophils, T cells, natural killer (NK) cells, B cells, and antigen-presenting cells. Cell homeostasis and apoptosis are altered (Clementi et al. 2021). Lung damage also occurs through the action of reactive oxygen and nitrogen species (Hartshorn, 2020).

There is a close relationship between IL-17, neutrophils and lung tissue. These cells perform phagocytosis in response to tissue damage, apoptotic and releasing repairing cytokines. IL-17 levels have been correlated with the severity of neutrophil infiltration (Sun et al. 2005; Oboki et al. 2008), suggesting that the expression of this cytokine may influence the pathogenesis or severity of the disease.

The pandemic group samples have a higher IL-17A tissue expression than the non-pandemic group (Table 1). Recently, Azevedo and colleagues (2020), describing a pediatric population that died from non-pandemic acute viral pneumonia compared to the non-viral pneumonia group, identified a higher level of IL-17A and IL-17RA in the former group. Particularly in the lung, responses to chemotactic signaling performed by IL-17A lead to the accumulation of neutrophils in the alveoli, the increase of pulmonary vascular permeability, and the damage to the alveolar epithelium with consequent impairment of respiratory function (Wang 2018). A study comparing lung samples from patients who died of SARS-CoV-2 and H1N1pdm09 showed greater septal and intra-alveolar neutrophils recruitment in the H1N1pdm09 group (Azevedo et al. 2021).

IL-17A participates in neutrophil migration and indirectly influences the intensity of the damage caused by this process. The relationship of this cytokine with the unfavorable outcome in viral infections is still controversial (Camp & Jonsson 2017). The IL-17RA has an essential function in this context. The transmembrane glycoprotein binds with low affinity to IL-17A, thus playing a pathogenic role in many inflammatory and autoimmune diseases such as rheumatoid arthritis (NCBI, 2020b). Knockout mice for the IL17RA gene had lower mortality and minor lung injury (Crowe et al. 2009).

The Th17 response, different from the Th1/Th2 axis, is present in bacterial and viral infection processes and is classically associated with recruiting neutrophils to combat and eliminate the microorganisms. Additionally, to the role of IL-17A associated with the defense system, some studies have indicated that this cytokine could be related to the induction of viral replication. These aspects could help understand these patients' propensity for unfavorable outcomes (Yuan et al., 2010; Xie et al. 2012). Most viruses in the non-pandemic group comprise the RNA-virus type, such as the FLUA. This aspect does not provide evidence to support the significant differences observed between the pandemic and non-pandemic groups (Table 1). The question that could arise is whether the presence of the H1N1pdm09 could stimulate a greater production of IL-17A or if IL-17A produced in response to initial viral infection could improve the viral replication process, which would lead the patient to the worst outcome.

The different SNPs in the IL17 gene have been recently explored, aiming to gain knowledge about their association with the risk of disease (Kawaguchi et al. 2006; Arisawa et al. 2008; Rasouli et al. 2013; Saraiva et al., 2013; Gao et al. 2020; Xie et al. 2019; Yang et al. 2020). These aspects could help understand these patients' propensity for unfavorable outcomes (Yuan et al., 2010; Xie et al. 2012).

Single nucleotide polymorphisms contribute to different outcomes between individuals and populations for the same disease (Ramírez-Bello & Jiménez-Morales 2017). To analyze the frequency of genotypes, the concept of risk was adopted.

The rs3819025 G/A is a genetic variant in non-coding DNA. This SNP presented higher AA genotypic frequency in the pandemic group, implying risk related to the A allele (*p* < 0.001). The frequency of the A allele in 1000 genomes in the European population is 5,77% (NCBI, 2022c). Studies have recently addressed the association of this marked with the most varied conditions. The A allele of rs3819025 was associated with an increased risk of renal impairment in Chinese children with purple Henoch-Schonlein. The GA + AA genotypes presented an approximately 1.8 times higher risk of proteinuria and/or hematuria in this population when compared to the GG genotype (Xu et al. 2016). A relevant observation was the absence of the GG genotype for this SNP in the pandemic group and low frequency in the non-pandemic and control group (Table 2). The G allele of this polymorphism in the wild allele and the loss of heterozygosity (LOH) observed could be due to an adaptive process like what can see in cancer, where LOH is favorable for neoplasia (Ali et al. 2017; Pinal-Fernandez et al. 2018). Therefore, LOH in our study could be favoring a risk phenotype for severe acute respiratory syndrome caused by H1N1pdm09.

A correlation between cytokine levels expression and their cytokine encoding genes genotypes could contribute to understanding the disease pathophysiology. The pandemic group showed higher tissue expression of IL-17A (Table 1). This result was observed in the IL17A SNP rs3819025 (G/A) (Table 3), establishing the A allele as a possible biomarker associated with the highest tissue expression of this interleukin.

Two polymorphisms of the IL17RA gene, rs2241044 A/C and rs2241043 C/T, showed a statistically significant association (Table 2).

The rs2241044 A/C is a genetic variant in non-coding DNA. The frequency of the C allele in 1000 genomes in the European population is 51,89% (NCBI, 2022d). The CC genotype was more frequent in the pandemic group compared to the non-pandemic group (*p* = 0.012) and control group (*p* = 0.001). The C allele of this SNP could confer risk for H1N1pdm09. One study evaluated Th17 axis-related polymorphisms, including IL17A, in patients with celiac disease and found no risk associations (Medrano et al. 2012).

The rs2241043 C/T is a genetic variant in non-coding DNA. The frequency of the T allele in 1000 genomes in the European population is 59,05% (NCBI, 2022e). The TT genotype was more frequent in the pandemic group compared to the non-pandemic group (*p* = 0.005) and control group (*p* = 0.001). The T allele of this SNP could confer risk for H1N1pdm09. For the T allele in rs2241043, there was an association with earlier death after hospitalization in the non-pandemic group (Fig. 1). One study evaluated polymorphisms of the IL-17 family and its receptors related to the risk of atherosclerosis and found no relationship with rs2241043 C/T (Wu et al. 2018).

Hence the influence of genetic factors has been demonstrated in the types of immune response triggered by the host; identifying genetic markers that contribute to an unfavorable outcome could be an alternative to understanding the pathophysiology of viral infections and identifying susceptible individuals.

The authors recognize the limitations inherent in the study. The sample in all groups can be considered small, but it represents the difficulty of recruiting patients to participate in the study. FFPE tissue samples to analyze IL-17A and IL-17RA tissue expressions represent a static moment at the patient's death and do not demonstrate retrospective data regarding clinical evolution. Furthermore, the age difference between the two groups may be a limiting factor in the interpretation of tissue expression of IL17A since age can modulate the expression of this interleukin. Thus, the higher expression of IL17A in the pandemic group may be a result of the H1N1 pandemic condition itself, as well as its older age in relation to the non-pandemic group.

Another limitation is that the resultant genetic polymorphisms may not represent causality in the association. There is still the possibility that these SNPs are in connection imbalance with other SNPs not addressed in the study. Still, concerning genetics, we need specific functional studies to confirm the causality associated with these polymorphisms.

## Conclusions

Although some SNPs studied have borderline significance, it is believed that the results of this article can be used to understand the influence of these genetic markers associated with the worst clinical outcome of patients with H1N1pdm09. One variant of *IL17A* (A allele of rs3819025) and two variants of *IL17RA* (C allele of rs2241044 and T allele of rs2241043) could be a worse prognosis biomarker. Increased tissue IL-17A expression and shorter survival time in the pandemic H1N1 group could help understand the role of the Th17 response and viral infections.

## Data Availability

The datasets during and/or analyzed during the current study are available from the corresponding author upon reasonable request.
